# 293. Association of a Hospital Stress Indicator with Device Associated Infections

**DOI:** 10.1093/ofid/ofae631.083

**Published:** 2025-01-29

**Authors:** Andrea Parriott, Nadia Barahmani, Erin Epson

**Affiliations:** California Department of Public Health, Richmond, CA; California Department of Public Health, Richmond, CA; California Department of Public Health, Richmond, CA

## Abstract

**Background:**

Hospital surges during the COVID-19 pandemic provided an opportunity to assess the effects of strained hospital resources on patient safety outcomes and identify predictors of resilience. We sought to examine the relationship between a composite indicator of hospital stress and device-associated infections (DAI) including central-line associated bloodstream infections (CLABSI) and catheter-associated urinary tract infections (CAUTI) in California acute-care hospitals.

**Figure:**
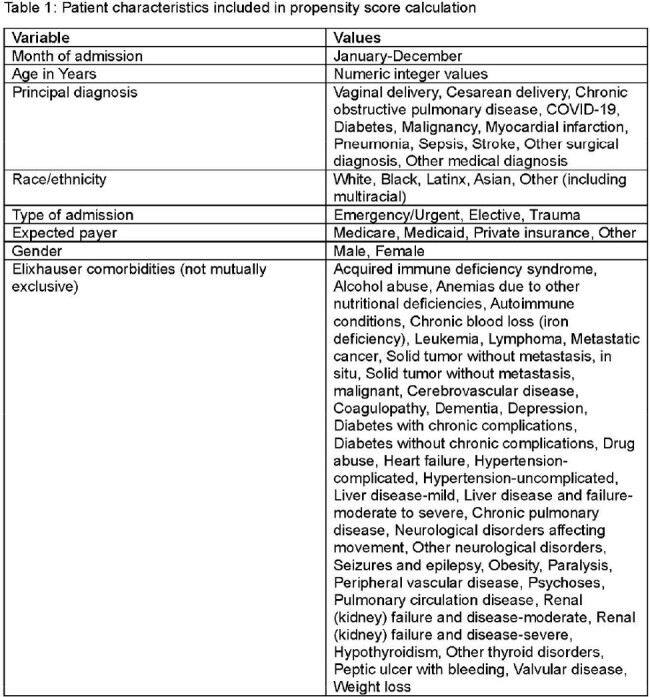

**Methods:**

We used data from the California Hospital Association daily survey to create a composite variable indicating hospital stress. Hospitals were considered stressed on a given day if they reported patients in surge beds, ventilated COVID-19 patients in the emergency department, or a critical staffing shortage. We matched data from the National Healthcare Safety Network (NHSN) on CLABSI and CAUTI occurring between June 2020 and December 2021 to inpatient discharge data from the California Office of Healthcare Access and Information (HCAI) by sex, birthdate, admission date, and hospital. Patients were considered exposed to hospital stress if the hospital was stressed on any day between their admission and infection onset or discharge. We used propensity score weighting for risk adjustment for patient characteristics including month of admission, age, sex, race, expected payer, principal diagnosis, type of admission (emergency/urgent, trauma, or elective) and comorbidities from linked HCAI records (Table 1). Odds ratios were calculated using logistic regression with the generalized estimating equation to account for clustering within facilities.

**Figure:**
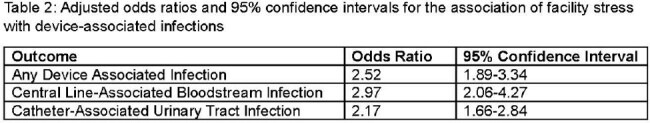

**Results:**

2,843 CLABSI and 2,870 CAUTI were successfully matched to patient records. Forty seven percent of patients were exposed to hospital stress. Risk-adjusted odds ratios were between 2 and 3 and significant at α=0.05 for all DAI, CLABSI, and CAUTI (Table 2).

**Conclusion:**

These findings validate that our measure of hospital stress was associated with increased risk of individual patient-level DAIs in 2020-2021. Further research will focus on generalizing results beyond 2020-2021 and assessing facility-level factors that mitigate the stress-patient outcome association.

**Disclosures:**

**All Authors**: No reported disclosures

